# PLK1 regulates hepatic stellate cell activation and liver fibrosis through Wnt/β‐catenin signalling pathway

**DOI:** 10.1111/jcmm.15356

**Published:** 2020-05-28

**Authors:** Yu Chen, Xin Chen, Ya‐Ru Ji, Sai Zhu, Fang‐Tian Bu, Xiao‐Sa Du, Xiao‐Ming Meng, Cheng Huang, Jun Li

**Affiliations:** ^1^ School of Pharmacy Anhui Key Laboratory of Major Autoimmune Diseases Anhui Institute of Innovative Drugs Anhui Medical University Hefei China; ^2^ The Key Laboratory of Anti‐inflammatory and Immune Medicines Anhui Medical University Ministry of Education Hefei China; ^3^ Institute for Liver Diseases of Anhui Medical University ILD‐AMU Anhui Medical University Hefei China; ^4^ Anhui Province Key Laboratory of Major Autoimmune Diseases Anhui Institute of Innovative Drugs First Affiliated Hospital Anhui Medical University Hefei China

**Keywords:** activation, HSCs, liver fibrosis, PLK1

## Abstract

As an outcome of chronic liver disease, liver fibrosis involves the activation of hepatic stellate cells (HSCs) caused by a variety of chronic liver injuries. It is important to explore approaches to inhibit the activation and proliferation of HSCs for the treatment of liver fibrosis. PLK1 is overexpressed in many human tumour cells and has become a popular drug target in tumour therapy. Therefore, further study of the function of PLK1 in the cell cycle is valid. In the present study, we found that PLK1 expression was elevated in primary HSCs isolated from CCl_4_‐induced liver fibrosis mice and LX‐2 cells stimulated with TGF‐β1. Knockdown of PLK1 inhibited α‐SMA and Col1α1 expression and reduced the activation of HSCs in CCl_4_‐induced liver fibrosis mice and LX‐2 cells stimulated with TGF‐β1. We further showed that inhibiting the expression of PLK1 reduced the proliferation of HSCs and promoted HSCs apoptosis in vivo and in vitro. Furthermore, we found that the Wnt/β‐catenin signalling pathway may be essential for PLK1‐mediated HSCs activation. Together, blocking PLK1 effectively suppressed liver fibrosis by inhibiting HSC activation, which may provide a new treatment strategy for liver fibrosis.

## 1 INTRODUCTION

Liver fibrosis is a consequence of the wound‐healing response to repeated liver injury characterized by excessive deposition of extracellular matrix (ECM) molecules.^1^, ^2^ There are various common causes of liver fibrosis, including hepatitis virus infection, alcoholic liver disease (ALD) or non‐alcoholic steatohepatitis (NASH), which may progress to liver cirrhosis and provide a pathological basis for the development of hepatocellular carcinoma (HCC).^3^, ^4^ The activation and proliferation of hepatic stellate cells (HSCs) induced by liver injury or micro‐environmental stimulation lead to the synthesis and secretion of a large number of ECM molecules and exert an important role in liver fibrogenesis.^4^ Given the important role of HSCs in liver fibrogenesis, a full review of the underlying molecular mechanisms is critical to determine new diagnostic and therapeutic targets for liver fibrosis. β‐catenin is the main downstream effector of the canonical Wnt/β‐catenin signalling pathway, and it activates target genes that are essential for proliferation and differentiation.^5^ The Wnt/β‐catenin pathway is an essential regulator of cell growth and proliferation, and it is important for normal liver development.^6^ Studies have confirmed that this pathway is closely related to the formation of HSCs and liver fibrosis.^7^, ^8^ However, the mechanisms involved in the activation of HSCs by the Wnt/β‐catenin pathway to participate in liver fibrosis remain unclear.

PLK1 (polo‐like kinase 1) belongs to the polo‐like kinase family and regulates cell mitosis, cytokinesis and DNA damage response.^9^, ^10^ Importantly, previous studies have identified that PLK1 is highly expressed in a variety of cancers including HCC, pancreatic carcinoma and renal cell carcinoma, and it is associated with decreased survival in cancer patients.^11^, ^12^ Several studies have shown that PLK1 is involved in the invasion and metastasis of cancer.^13^, ^14^ Furthermore, blocking the expression of PLK1 can effectively inhibit the proliferation of tumour cells and induce their apoptosis.^15^ In addition, PLK1 has been demonstrated as a marker associated with the cell cycle in acute idiopathic pulmonary fibrosis (IPF) and is a therapeutic target.^16^ Considering the above findings, we speculated whether PLK1 can regulate the proliferation and apoptosis of HSCs to promote the resolution of liver fibrosis. Interestingly, a previous study identified that PLK1 phosphorylation of axin2 facilitated the GSK3‐dependent phosphorylation of β‐catenin by enhancing binding between GSK3 and β‐catenin, offering a novel PLK1‐Wnt/β‐catenin signalling axis to treat prostate cancer.^17^, ^18^ In our study, we investigated whether PLK1 regulates HSCs via the Wnt/β‐catenin signalling pathway to influence the development of liver fibrosis.

To the best of our knowledge, this is the first study to demonstrate the essential role of PLK1 in the pathogenesis of liver fibrosis and identify the potential mechanisms involved. We found that reduced PLK1 expression effectively prevented HSC activation. In addition, we revealed that inhibition of PLK1 promoted HSC apoptosis and reduced liver fibrosis via the Wnt/β‐catenin signalling pathway in vivo and in vitro.

## 2 MATERIALS AND METHODS

### 2.1 Mouse model of liver fibrosis

All experiments were conducted as required of the Ethics Committee and Animal Experimental Committee at Anhui Medical University. Male C57BL/6J mice (8 weeks of age) were purchased from the Animal Experiment Center of Anhui Medical University. C57BL/6J mice were intraperitoneally injected twice a week with a 10% solution of CCl_4_ in olive oil or olive oil alone (vehicle control) at a dose of 0.001 mL/g for 6 weeks. Mice were killed 3 days after the last injection.

### 2.2 Adeno‐associated virus (AAV) infection

Purified adeno‐associated viral vector serotype 8 (AAV8) encoding PLK1 was generated by Hanheng Biotechnology (Shanghai, China). C57BL/6J mice received a single tail vein injection of AAV8 encoding PLK1 at a concentration of 1 × 10^12^ vg/mL. The transfection efficiency was measured by Western blotting and real‐time PCR analysis.

### 2.3 Human samples

Ten normal and fourteen human liver fibrosis samples were received from the First Affiliated Hospital of Anhui Medical University (Anhui, China). All specimens were obtained after receiving informed consent from each patient, and the experiment was performed in accordance with the Declaration of Helsinki and with approval by the Ethics Committee of Anhui Medical University. Part of each sample was fixed in 4% paraformaldehyde and embedded in paraffin, whereas the rest was stored at −80°C.

### 2.4 Isolation of primary HSCs

HSCs were isolated as previously described^19^, ^20^ with some modifications. Briefly, mouse primary HSCs were isolated employing a two‐step collagenase (Sigma)‐pronase (Sigma) perfusion of mouse livers, followed by OptiPrep density gradient centrifugation (Axis Shield, Norway). Finally, the expression level of α‐SMA (an HSC marker) was detected by Western blotting and real‐time PCR.

### 2.5 Histology and immunohistochemistry

A after 4% paraformaldehyde fixation and paraffin embedding, liver tissues were sectioned at 8 µm thickness for haematoxylin and eosin staining (H&E), Sirius red staining and immunohistochemical staining of α‐SMA (1:400; Abcam, USA) and PLK1 (1:400; Abcam, USA) to examine liver pathology. We employed a microwave‐based antigen retrieval technique following standard methods.^21^ These sections were examined by a digital slide scanner (3DHISTECH, Hungary).

### 2.6 Immunofluorescence staining

Frozen liver tissue sections were blocked with 10% bovine serum albumin (BSA) at room temperature for 1 hour. Anti‐PLK1 (1:200; Abcam, USA) and anti‐α‐SMA (1:200; Abcam, USA) antibodies were added to the sections and incubated at 4°C for 16 hour. Sections were then incubated with a combination of TRITC‐conjugated (1:100, ZSGB‐Bio, China) and FITC‐conjugated (1:100, ZSGB‐Bio, China) secondary antibodies at room temperature for 60 mins in the dark. The positive expression of α‐SMA and PLK1 was observed by inversion fluorescence microscopy.

### 2.7 Serum biochemical value analysis

Serum was collected from whole blood samples by centrifugation at 1000 g for 30 minutes at room temperature. Serum alanine aminotransferase and aspartate aminotransferase (ALT and AST) were measured using an ALT and AST Assay Kit (Jiancheng Bioengineering Institute, Nanjing, China) according to the manufacturer's instructions.

### 2.8 Western blot analysis

Proteins samples were extracted from primary HSCs and LX‐2 cells using RIPA lysis buffer (Beyotime, China). Equal amounts of proteins were separated by 10% SDS‐PAGE and transferred onto PVDF membranes (Millipore, USA). The membranes were blocked with 5% skim milk for 1 hour at room temperature and then incubated with primary antibodies overnight at 4°C. The following antibodies were used for Western blotting: β‐actin (1:500; Bioss, China), Col1α1 (1:500; Bioss), α‐SMA (1:500; Bioss), PLK1 (1:1000; Abcam), Bax (1:800; Abcam), Bcl‐2 (1:800; Abcam), cleaved caspase‐3 (1:1000; Abcam), β‐catenin (1:500, Bioss), c‐Myc (1:500; Bioss) and Cyclin D1 (1:500; Bioss). The membranes were incubated with HRP‐conjugated secondary antibodies (1:10000; ZSGB‐Bio, China) for 60 minutes at room temperature. The protein bands were detected with a chemiluminescent (ECL) system (Bio‐Rad, USA) and analysed using IMAGE J software (National Institutes of Health, USA).

### 2.9 Real‐time PCR analysis

Total RNA was extracted from primary HSCs or LX‐2 cells using the TRIzol reagent (Invitrogen, USA) following the manufacturer's protocols. Quantitative detection was performed using a Spectrophotometer NanoDrop 2000 (Thermo Scientific, USA), and cDNA was synthesized using a PrimeScript™RT Master Mix (Takara, Japan). Real‐time PCR samples were run on the CFX96 Real‐Time PCR Detection System (Bio‐Rad, USA) for 10 minutes at 95°C, followed by 40 cycles at 95°C for 15 seconds and at 60°C for 1 minute. GAPDH expression was used as an internal control. The sequences of the primers for real‐time PCR are listed in Table 1.

**TABLE 1:** Primers sequences used for real‐time PCR

Gene	Forward(5′‐3′)	Reverse(5′‐3′)
*Mouse*		
PLK1	CTCCCTTTGAGACCTCGTGC	TGGGATGGTGAGGCAGGTAA
α‐SMA	CGGGAGAAAATGACCCAGATT	AGGGACAGCACAGCCTGAATAG
Col1α1	GGAGAGTACTGGATCGACCCTAAC	ACACAGGTCTGACCTGTCTCCAT
TIMP‐1	GCAACTCGGACCTGGTCATAA	CGGCCCGTGATGAGAAACT
GAPDH	GGACCTCATGGCCTACATGG	TAGGGCCTCTCTTGCTCAGT
*Human*		
PLK1	CCATCACCTGCCTGACCATT	CCTCACCTGTCTCTCGAACC
α‐SMA	ATCAAGGAGAAACTGTGTTATGTAG	GATGAAGGATGGCTGGAACAGGGTC
Col1α1	TCTAGACATGTTCAGCTTTGTGGAC	TCTGTACGCAGGTGATTGGTG
TIMP‐1	CTTCTGCAATTCCGACCTCGT	ACGCTGGTATAAGGTGGTCTG
β‐actin	GCCAACACAGTGCTGTCTGG	CTCAGGAGGAGCAATGATCTTG

### 2.10 Cell culture

LX‐2 cells were cultured in DMEM (Gibco, USA) supplemented with 10% foetal bovine serum (Gibco, USA), 100 U/mL penicillin and 100 mg/mL streptomycin, and incubated in a 5% CO_2_ incubator at 37°C.

### 2.11 Transfection with PLK1 plasmid in vitro

To overexpress PLK1, LX‐2 cells were transiently transfected with pLenO‐GTP‐3XFLAG‐PLK1 (Ruan Tuo, China) using Lipofectamine™ 2000 (Invitrogen, USA) according to the manufacturer's guidelines. LX‐2 cells were treated with an empty plasmid as a negative control. The transfection efficiency was measured by Western blotting and real‐time PCR analysis.

### 2.12 Transfection with PLK1 CRISPR/Cas9 lentivirus in vitro

A validated human PLK1 CRISPR/Cas9 KO lentivirus was purchased from Hanheng Biotechnology (Shanghai, China). The PLK1‐specific gRNA sequence was 5’‐CACCGTGCCAAGTGCTTCGAGATCT‐3’. Briefly, LX‐2 cells were transferred to 12‐well plates. After 24 hours, the cells had reached 60%‐70% confluency and were transfected with PLK1 CRISPR/Cas9 lentivirus according to the manufacturer's protocol. The transfection efficiency was measured by Western blotting and real‐time PCR analysis.

### 2.13 Cell proliferation assay

The proliferation of LX‐2 cells was determined by a Cell Counting Kit‐8 (CCK‐8) (Best Bio, China). Briefly, the transfected cell suspensions were collected, added to 96‐well plates and incubated at 37°C overnight. Then, 10 µL of the CCK‐8 solution was added to each well for 3 hours, and the absorbance value in each well at 450 nm was detected using a microplate reader (Bio‐Tek EL, USA).

### 2.14 Cell apoptosis analysis

The apoptosis of LX‐2 cells was analysed by flow cytometry using the Annexin V‐FITC/PI Apoptosis Kit (Best Bio, China). Cells were centrifuged and suspended in 400 µL Annexin binding solution. Then, 5 µL Annexin V‐FITC staining solution was added to the suspension and incubated for 15 minutes followed by the addition of PI and incubated for 5 minutes in the dark at 4°C before flow cytometry analysis (Beckman Coulter, USA).

### 2.15 Cell cycle analysis

The amount of DNA present in LX‐2 cells was assessed by the Cell Cycle Analysis Kit (Best Bio, China). The cells were collected by centrifugation, fixed with cold ethanol overnight at 4°C, and then centrifuged and washed. The cells were suspended in 500 µL cold PBS with 20 µL RNase A solution at 37°C for 30 minutes. After filtration using a 400‐mesh screen, the cells were resuspended in 400 µL PI and incubated at 4°C in the dark for 30 minutes. Finally, the cells were analysed by flow cytometry (Beckman Coulter, USA).

### 2.16 Statistical analysis

Data collected from this study were analysed using one‐way analysis of variance (ANOVA), followed by Newman‐Keuls post hoc test (Prism 5.0 GraphPad software, USA), and are expressed as the mean ± SEM.

## 3 RESULTS

### 3.1 PLK1 was highly expressed in human liver fibrosis tissues

**FIGURE 1:**
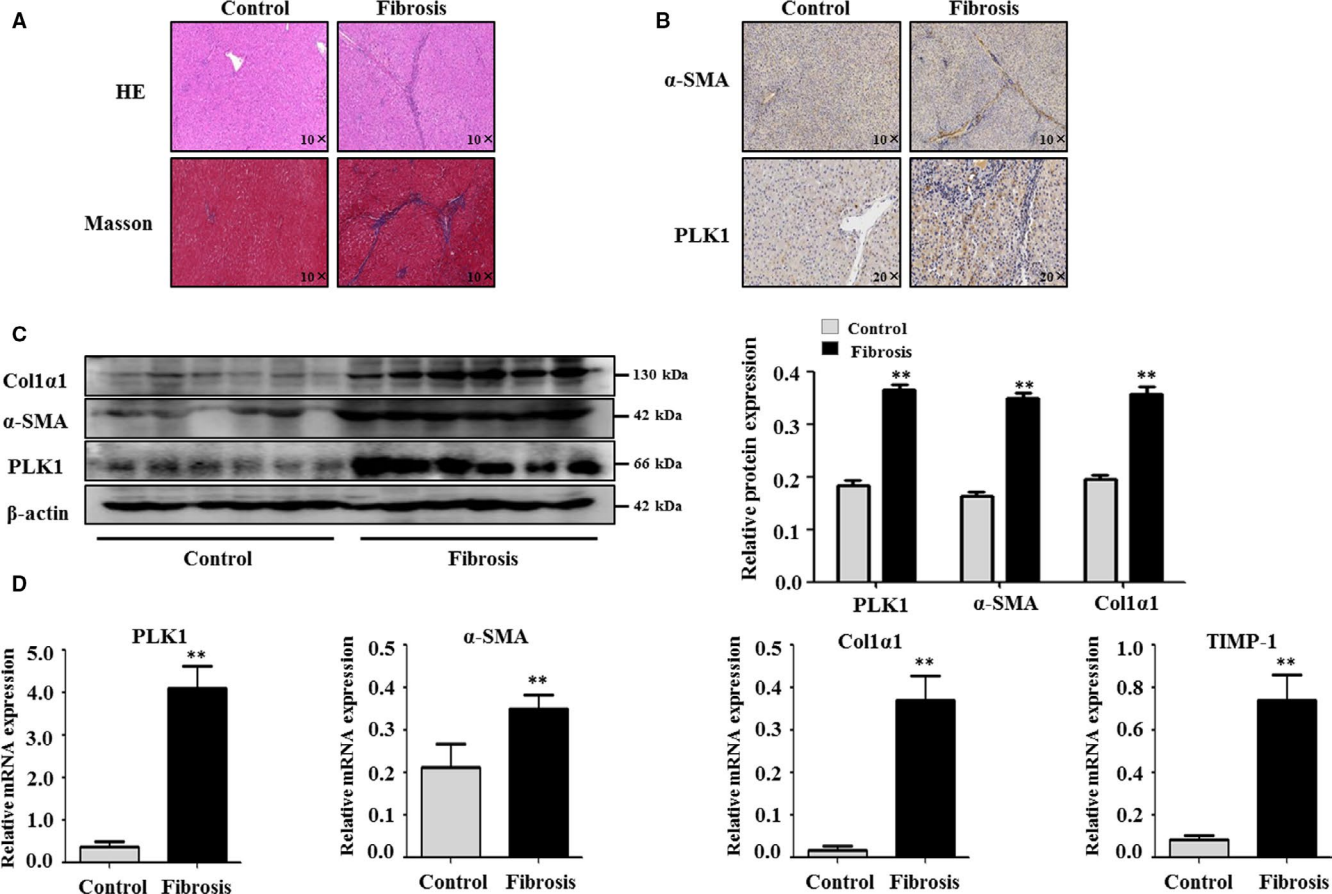
PLK1 was more highly expressed in human liver fibrosis. A, Representative sections of haematoxylin and eosin (H&E) and Masson staining (B) and IHC of α‐SMA and PLK1 in human liver fibrosis and healthy control samples. C, Protein expression of PLK1, α‐SMA and Col1α1 was analysed by Western blotting of human fibrotic liver and healthy liver samples. D, PLK1, α‐SMA, Col1α1 and TIMP‐1 mRNA levels were analysed by real‐time PCR in human liver fibrosis samples. The data represent the mean ± SEM of at least three independent experiments. **P < 0.01 vs healthy controls

We analysed PLK1 levels in human healthy control and liver fibrosis tissue. The pathological results showed that the liver tissues were structurally disordered and the fibrosis tissues were significantly hyperplastic, as shown by H&E staining in patients with liver fibrosis (Figure 1A). The fibrotic parts were indicated by Sirius red staining (Figure 1A), and activated HSCs were identified by α‐SMA–positive areas (Figure 1B). Immunohistochemical analyses revealed low expression of PLK1 in healthy livers, whereas much higher expression of PLK1 was evident in the perivascular region of the portal vein and lesion boundary in patients with liver fibrosis (Figure 1B). Protein expression of PLK1, α‐SMA and Col1α1 was elevated in human liver fibrosis compared with healthy livers by Western blotting (Figure 1C). Similarly, the mRNA levels of PLK1 and fibrogenic genes (α‐SMA, Col1α1 and TIMP‐1) were higher in liver fibrosis than in healthy livers, as shown by real‐time PCR (Figure 1D). These results indicate that the expression of PLK1 is elevated in liver fibrosis, and that up‐regulated PLK1 expression is associated with liver fibrosis.

### 3.2 Up‐regulation of PLK1 expression in HSCs is associated with CCl_4_‐induced liver fibrosis

To evaluate whether PLK1 expression is correlated with liver fibrosis, we developed a CCl_4_‐induced mouse liver fibrosis model for histopathological studies. Liver injury was observed in liver fibrosis mice by H&E staining (Figure S1A). The collagen area was indicated by Sirius red staining, and the activated HSCs were highlighted by α‐SMA positivity (Figure S1A). Immunohistochemical analyses demonstrated that PLK1 expression was increased in CCl_4_‐induced mice compared with vehicle mice (Figure S1A). The α‐SMA–positive and PLK1‐positive area quantitative results are shown in Figure S1A. Serum levels of AST and ALT were higher in CCl_4_‐induced liver fibrosis mice compared with vehicle mice (Figure S1B). Furthermore, we found an increase in PLK1, α‐SMA and Col1α1 mRNA and protein levels in primary HSCs isolated from CCl_4_‐induced liver fibrosis mice compared with vehicle mice (Figure S1C,D). We performed co‐localization studies of PLK1 with α‐SMA by immunofluorescence double staining in vivo. Interestingly, the results revealed a dramatic up‐regulation of PLK1 (red) expression in the activated HSCs that were positive for α‐SMA (green) after the induction of liver fibrosis by CCl_4_ (Figure S1E). The co‐localization positive area quantitative results are shown in Figure S1E. These studies demonstrate that PLK1 expression is increased in the liver fibrosis mouse model, which is consistent with the results in humans, suggesting that PLK1 may be related to HSCs activation in liver fibrosis.

### 3.3 Inhibition of PLK1 alleviates CCl_4_‐induced liver fibrosis

**FIGURE 2:**
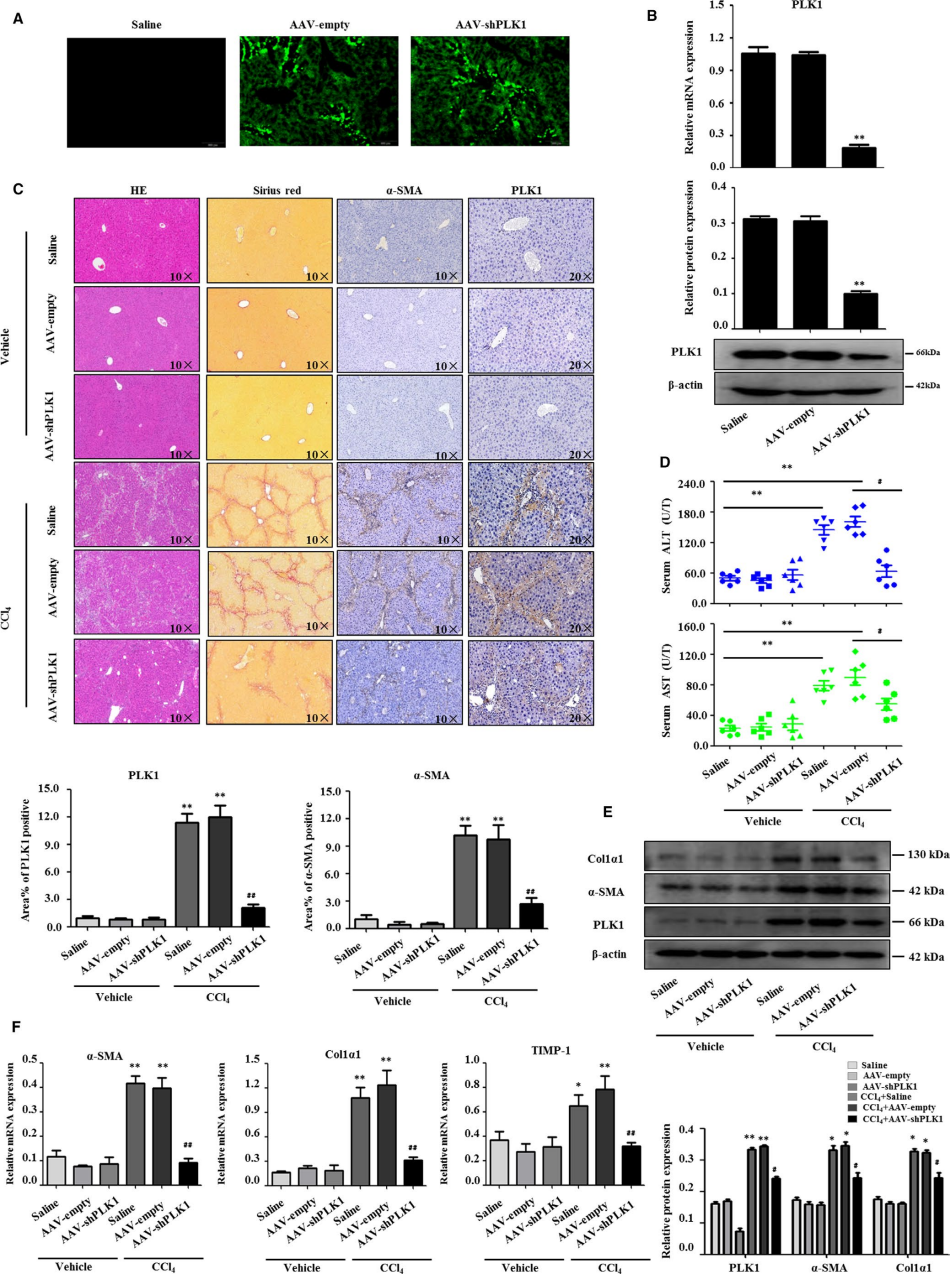
Inhibition of PLK1 alleviates CCl_4_‐induced liver fibrosis. A, C57/BL6 mice were injected with AAV‐shPLK1 virus via the tail vein; the efficiency of AAV‐mediated PLK1 decreases in mice was monitored by the co‐expressed marker of GFP. B, Western blot analysis and real‐time PCR showed PLK1 expression decreased with AAV‐shPLK1 administration. C, H&E staining and Sirius red staining in CCl_4_‐treated mice following AAV‐shPLK1 delivery, and IHC of α‐SMA and PLK1. Representative images are presented. Scale bar, 100 µmol/L and 50 µmol/L. Quantification of fibrosis based on immunohistochemistry analysis of α‐SMA and PLK1. D, Measurement of serum ALT and AST from mice in CCl_4_‐treated mice following AAV‐shPLK1 delivery. E, Protein expression of PLK1, α‐SMA and Col1α1, (F) and mRNA levels of α‐SMA, Col1α1 and TIMP‐1 were attenuated in mice treated with AAV‐shPLK1. Data shown are the mean ± SEM of three independent experiments. *P < 0.05, **P < 0.01 vs saline; ^#^ *P* < 0.05, ^##^ *P* < 0.01 vs CCl_4_ + AAV‐empty

To define the role of PLK1 in liver fibrogenesis, we investigated the functional effects of PLK1 on liver fibrosis in CCl_4_‐induced liver fibrosis mice in vivo. Mice were injected with adeno‐associated virus (AAV)‐shRNA‐PLK1 to knockdown PLK1 expression in the liver, and mice injected with an empty AAV vector were used as controls (Figure 2A). Mice were repetitively treated with CCl_4_ for 6 weeks. We detected the knockdown efficiency of AAV‐shPLK1 on PLK1 by Western blotting and real‐time PCR (Figure 2B). Inhibition of PLK1 alleviated CCl_4_‐induced liver injury and fibrosis compared with AAV‐empty–treated mice by H&E and Sirius red staining (Figure 2C). Consistently, IHC staining results showed that the decrease in PLK1 significantly reduced α‐SMA expression (Figure 2C). The quantification of α‐SMA–positive and PLK1‐positive areas is shown in Figure 2C. Furthermore, there was a common reduction in the levels of ALT and AST in serum from CCl_4_‐induced liver fibrosis mice injected with AAV‐shPLK1 compared with the mice that received AAV‐empty (Figure 2D). Moreover, low expression of PLK1 by AAV‐shPLK1 led to a significant decrease in the expression of α‐SMA and Col1α1 protein in HSCs from CCl_4_‐induced liver fibrosis mice (Figure 2E). The mRNA levels of α‐SMA, Col1α1 and TIMP‐1 were notably attenuated in CCl_4_‐treated PLK1 knockdown mice compared with AAV‐empty mice (Figure 2F). Collectively, these observations revealed that inhibition of PLK1 alleviates liver fibrosis and the activation of HSCs in vivo.

### 3.4 Blocking PLK1 attenuates the activation of LX‐2 cells stimulated with TGF‐β1

**FIGURE 3:**
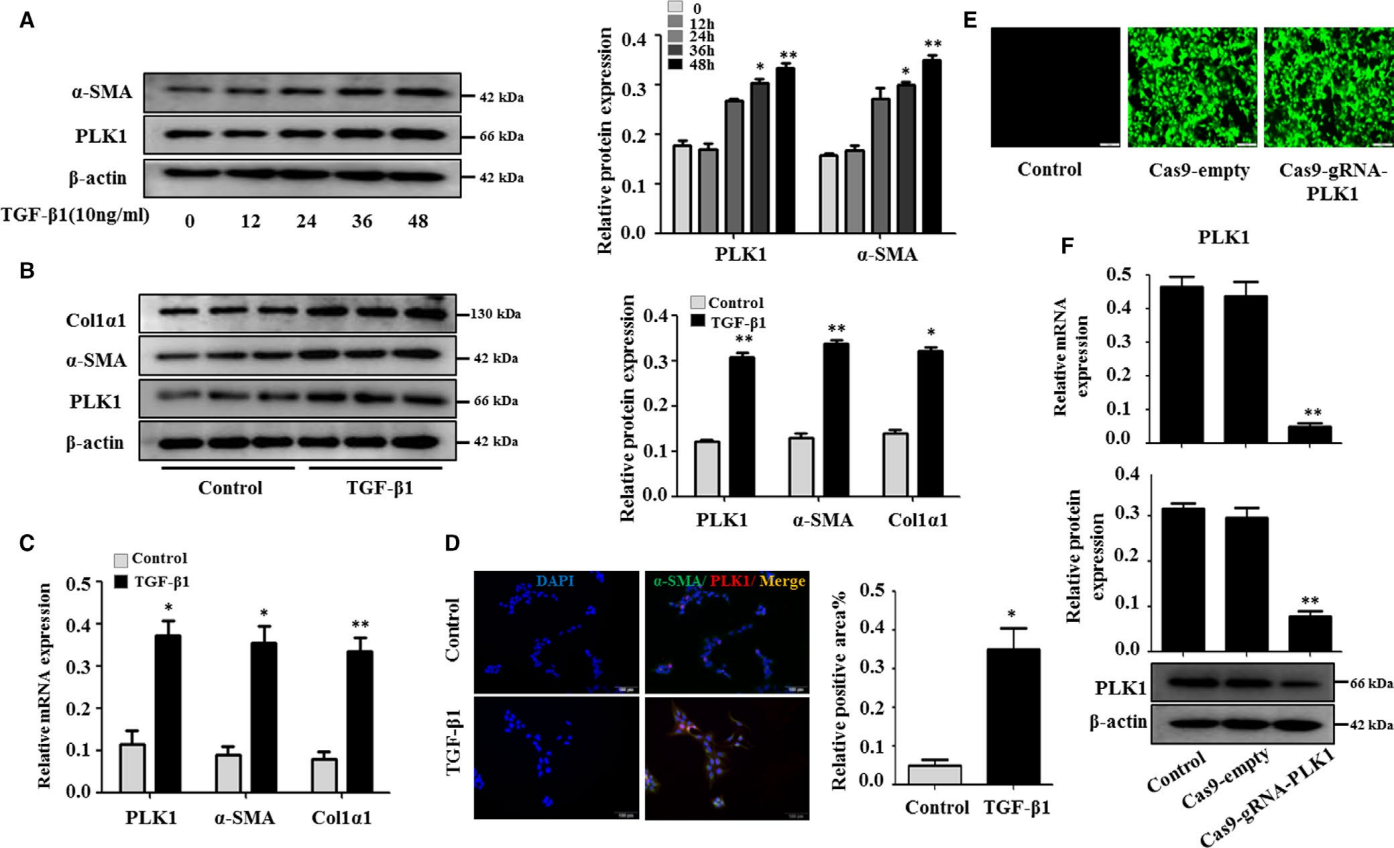
Up‐regulation of PLK1 expression in LX‐2 cells stimulated with TGF‐β1. A, LX‐2 cells were activated by TGF‐β1 (10 ng/mL) for 0, 12, 24, 36 and 48 h, and the protein expression of PLK1 and α‐SMA was examined. *P < 0.05, **P < 0.01 vs 0 h. (B,C) Protein and mRNA expression of PLK1, α‐SMA and Col1α1 in LX‐2 cells treated with TGF‐β1 (10 ng/mL) for 48 h was assessed. *P < 0.05, **P < 0.01 vs control. D, Representative double immunofluorescence images of α‐SMA (green) and PLK1 (red) in LX‐2 cells are presented. Scale bar, 100 µmol/L. Quantitative results of positive co‐localization areas are shown. E, LX‐2 cells were transfected with Cas9‐gRNA‐PLK1 lentivirus, and the efficiency of PLK1 reduction in LX‐2 cells was monitored by co‐expression of GFP. F, Western blot and real‐time PCR analyses of PLK1 from LX‐2 cells transfected with Cas9‐gRNA‐PLK1 or Cas9‐empty. *P < 0.05, **P < 0.01 vs control. Data shown are the mean ± SEM from three independent experiments

**FIGURE 4:**
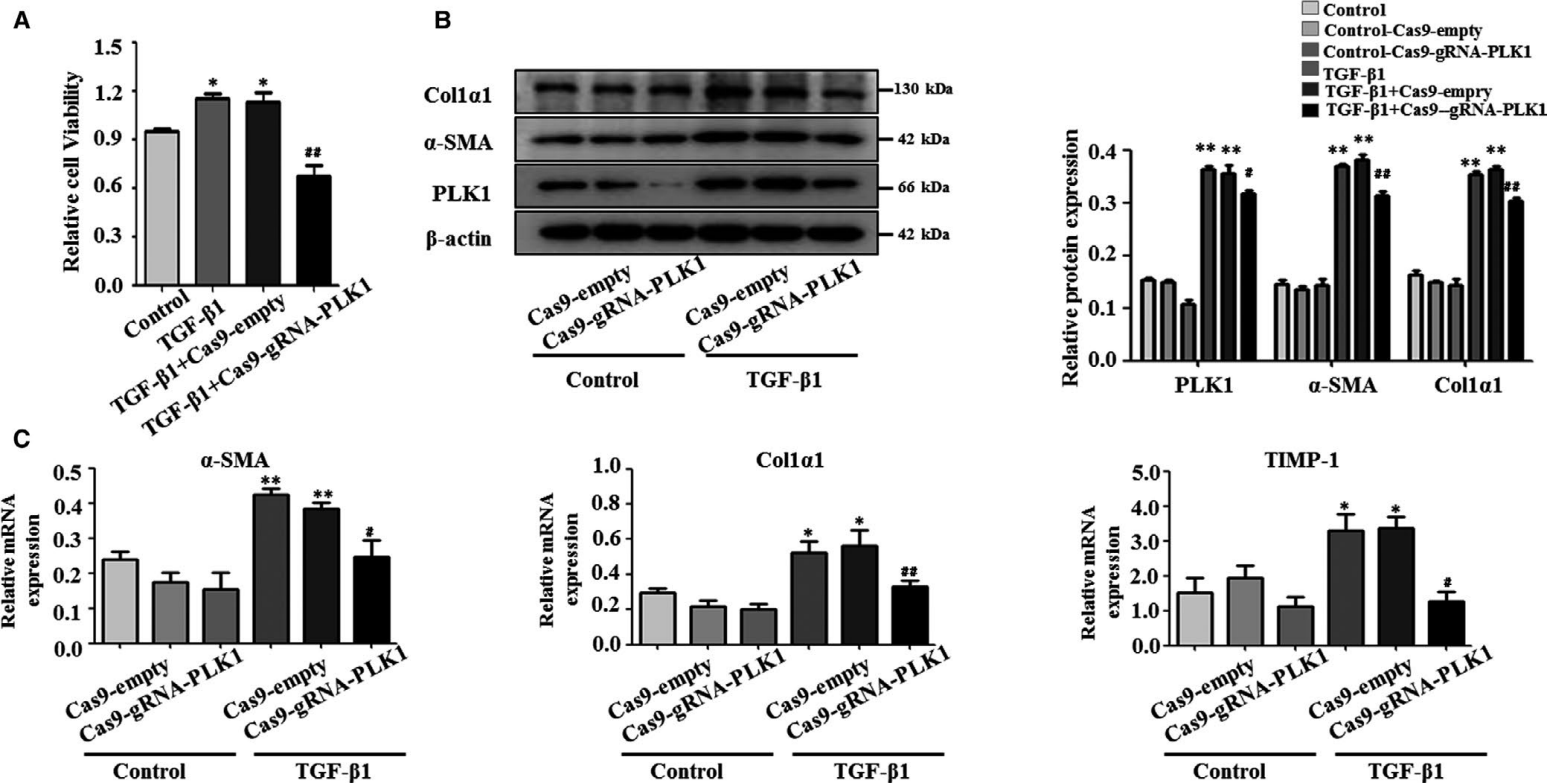
Blocking PLK1 attenuates the activation of LX‐2 cells stimulated with TGF‐β1. A, Relative cell viability of LX‐2 cells was measured by CCK‐8 assay. B, Western blot analyses of PLK1, α‐SMA and Col1α1, (C) and mRNA levels of α‐SMA, Col1α1 and TIMP‐1 in LX‐2 cells stimulated with TGF‐β1 following transduction with Cas9‐gRNA‐PLK1. *P < 0.05, **P < 0.01 vs control; ^#^ *P* < 0.05, ^##^ *P* < 0.01 vs TGF‐β1 + Cas9‐empty. Data shown are the mean ± SEM from three independent experiments

To further assess whether PLK1 expression is related to HSCs activation in liver fibrosis, we investigated the functional effects of silencing PLK1 on liver fibrosis in LX‐2 cells in vitro. We found that α‐SMA and PLK1 expression levels were enhanced in a time‐dependent manner by Western blotting in LX‐2 cells stimulated with TGF‐β1 (Figure 3A). Therefore LX‐2 cells were treated with 10 ng/mL TGF‐β1 for 48 hour in the following experiments. Protein expression and mRNA levels of PLK1, α‐SMA and Col1α1 were enhanced compared with the controls by Western blotting and real‐time PCR (Figure 3B,C). PLK1 expression was also increased in LX‐2 cells stimulated with TGF‐β1 (10 ng/mL), as determined by immunofluorescence staining (Figure 3D). We used clustered regularly interspaced short palindromic repeat (CRISPR)‐CRISPR‐associated protein 9 (CRISPR/Cas9) to silence PLK1 by co‐expressing PLK1‐specific RNA and Cas9 endonuclease. The green fluorescent protein (GFP) signal in the LX‐2 cells transfected with Cas9‐gRNA‐PLK1 lentivirus showed that the cells were successfully infected (Figure 3E). After the transfection of Cas9‐gRNA‐PLK1 into LX‐2 cells, the efficiency of PLK1 silencing was measured and confirmed by Western blotting and real‐time PCR (Figure 3F). Inhibition of PLK1 significantly reduced the viability of LX‐2 cells stimulated with TGF‐β1 by CCK‐8 analysis (Figure 4A). After the transfection of Cas9‐gRNA‐PLK1 into LX‐2 cells, protein expression of α‐SMA and Col1α1 was remarkably down‐regulated compared with that of the Cas9‐empty group (Figure 4B). Consistently, treatment of LX‐2 cells with Cas9‐gRNA‐PLK1 significantly reduced the mRNA levels of α‐SMA, Col1α1 and TIMP‐1 compared with the Cas9‐empty group (Figure 4C). These results indicated that PLK1 was enhanced in LX‐2 cells stimulated with TGF‐β1 and that blocking PLK1 attenuated the activation of these TGF‐β1–treated LX‐2 cells.

### 3.5 PLK1 promotes the activation of LX‐2 cells stimulated with TGF‐β1

Next, LX‐2 cells were transfected with GTP‐PLK1 plasmid, and the efficiency of PLK1 overexpression was measured by Western blotting (Figure S2A). PLK1 overexpression significantly increased LX‐2 cell viability by CCK‐8 analysis compared with the GTP‐empty plasmid‐transfected group (Figure S2B). We identified that the expression levels of α‐SMA and Col1α1 were significantly up‐regulated in LX‐2 cells following overexpression of the GTP‐PLK1 plasmid compared with the GTP‐empty group (Figure S2C). Similarly, PLK1 overexpression markedly increased the mRNA levels of α‐SMA, Col1α1 and TIMP‐1 compared with the GTP‐empty plasmid‐transfected group (Figure S2D). These results indicate that PLK1 promotes the activation of LX‐2 cells stimulated by TGF‐β1 in vitro.

### 3.6 Inhibition of PLK1 facilitates apoptosis of HSCs in liver fibrosis

**FIGURE 5:**
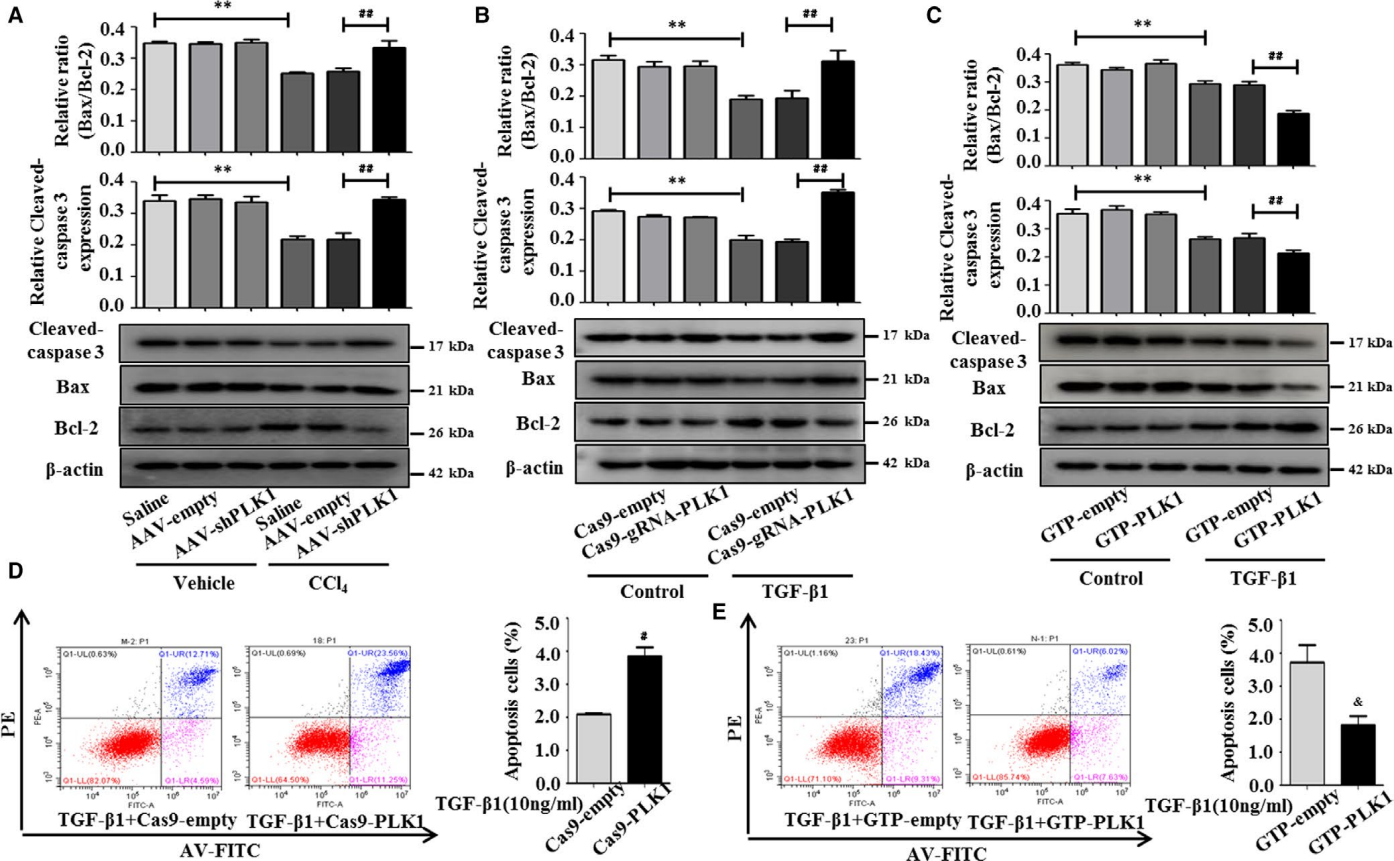
Inhibition of PLK1 facilitates apoptosis of HSCs in liver fibrosis. A, Expression of apoptosis‐associated proteins (Bax, Bcl‐2 and cleaved caspase‐3) in mice injected with AAV‐shPLK1 virus. *P < 0.05, **P < 0.01 vs saline; ^#^ *P* < 0.05, ^##^ *P* < 0.01 vs CCl_4_ + AAV‐empty. (B,C) Expression of apoptosis‐associated proteins (Bax, Bcl‐2 and cleaved caspase‐3) in LX‐2 cells transfected with Cas9‐gRNA‐PLK1 or GTP‐PLK1. *P < 0.05, **P < 0.01 vs control; ^#^ *P* < 0.05, ^##^ *P* < 0.01 vs TGF‐β1 + Cas9‐empty; ^&^ *P* < 0.05, ^&&^ *P* < 0.01 vs TGF‐β1 + GTP‐empty. D, Effect of decreased PLK1 on the apoptosis of TGF‐β1–activated LX‐2 cells was determined by flow cytometry. ^#^ *P* < 0.05 vs TGF‐β1 + Cas9‐empty. E, Effect of up‐regulated PLK1 on the apoptosis of TGF‐β1–activated LX‐2 cells was assessed by flow cytometry. ^&^ *P* < 0.05 vs TGF‐β1 + GTP‐empty. Data shown are the mean ± SEM, and representative images of three independent experiments are shown

To verify the effect of PLK1 knockdown on the apoptosis of HSCs, we detected the expression of cleaved caspase‐3, Bcl‐2 and Bax by Western blot analysis. The results showed that the expression of cleaved caspase‐3 was increased and that the Bax/Bcl‐2 ratio was enhanced in HSCs from CCl_4_‐induced liver fibrosis mice injected with AAV‐shPLK1 compared with the AAV‐empty group (Figure 5A). Consistently, the expression of cleaved caspase‐3 and the Bax/Bcl‐2 ratio was increased compared with the Cas9‐empty group after the transfection of LX‐2 cells with Cas9‐gRNA‐PLK1 (Figure 5B). In contrast, we identified that the expression of cleaved caspase‐3 and the Bax/Bcl‐2 ratio were significantly down‐regulated in LX‐2 cells following GTP‐PLK1 overexpression compared with the GTP‐empty group (Figure 5C). In addition, PLK1 silencing increased the percentage of apoptotic LX‐2 cells, whereas overexpression of PLK1 reduced the percentage of apoptotic LX‐2 cells by flow cytometry (Figure 5D,E). Taken together, our analyses suggest that inhibiting PLK1 promotes the apoptosis of HSCs by mitochondrial‐ and caspase‐dependent pathways in vivo and in vitro, providing evidence that inhibiting PLK1 ameliorates liver fibrosis.

### 3.7 PLK1 promotes the proliferation of HSCs in liver fibrosis by regulating the Wnt/β‐catenin signalling pathway

**FIGURE 6:**
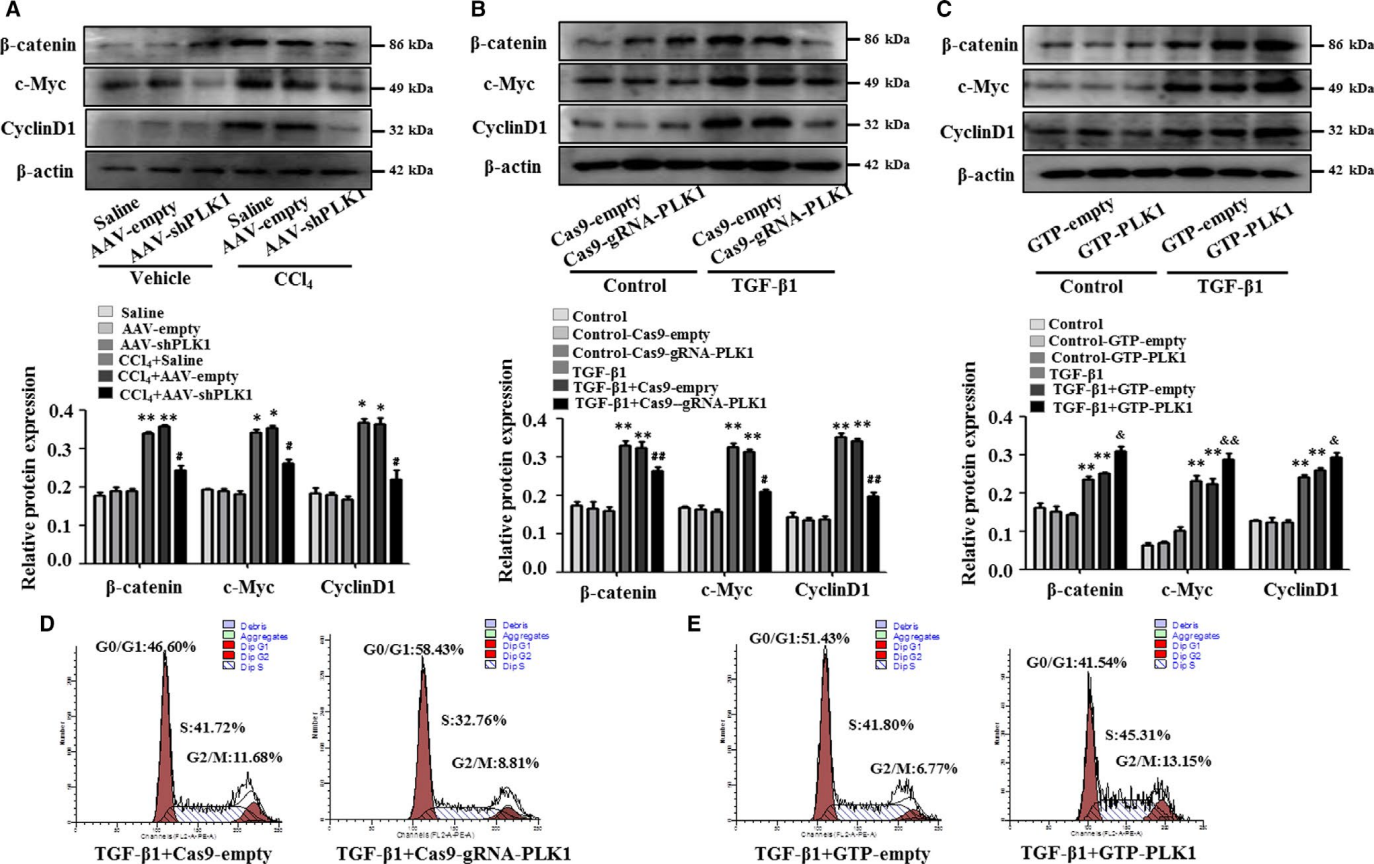
PLK1 promotes the proliferation of HSCs in liver fibrosis. A, β‐catenin, c‐Myc and Cyclin D1 expression in primary HSCs isolated from mice injected with AAV‐shPLK1 virus by Western blotting. *P < 0.05, **P < 0.01 vs saline; ^#^ *P* < 0.05, ^##^ *P* < 0.01 vs CCl_4_ + AAV‐empty. (B,C) β‐catenin, c‐Myc and Cyclin D1 expression was assessed in LX‐2 cells transfected with Cas9‐gRNA‐PLK1 or GTP‐PLK1. *P < 0.05, **P < 0.01 vs control; ^#^ *P* < 0.05, ^##^ *P* < 0.01 vs TGF‐β1 + Cas9‐empty; ^&^ *P* < 0.05, ^&&^ *P* < 0.01 vs TGF‐β1 + GTP‐empty. D, Flow cytometry analysis was performed to examine the cell cycle distribution of TGF‐β1–activated LX‐2 cells transfected with Cas9‐gRNA‐PLK1. E, Effect of up‐regulated PLK1 on the cell cycle of TGF‐β1–activated LX‐2 cells transfected with GTP‐PLK1 by flow cytometry analysis. Representative images of at least three independent experiments. Data shown are the mean ± SEM

**FIGURE 7:**
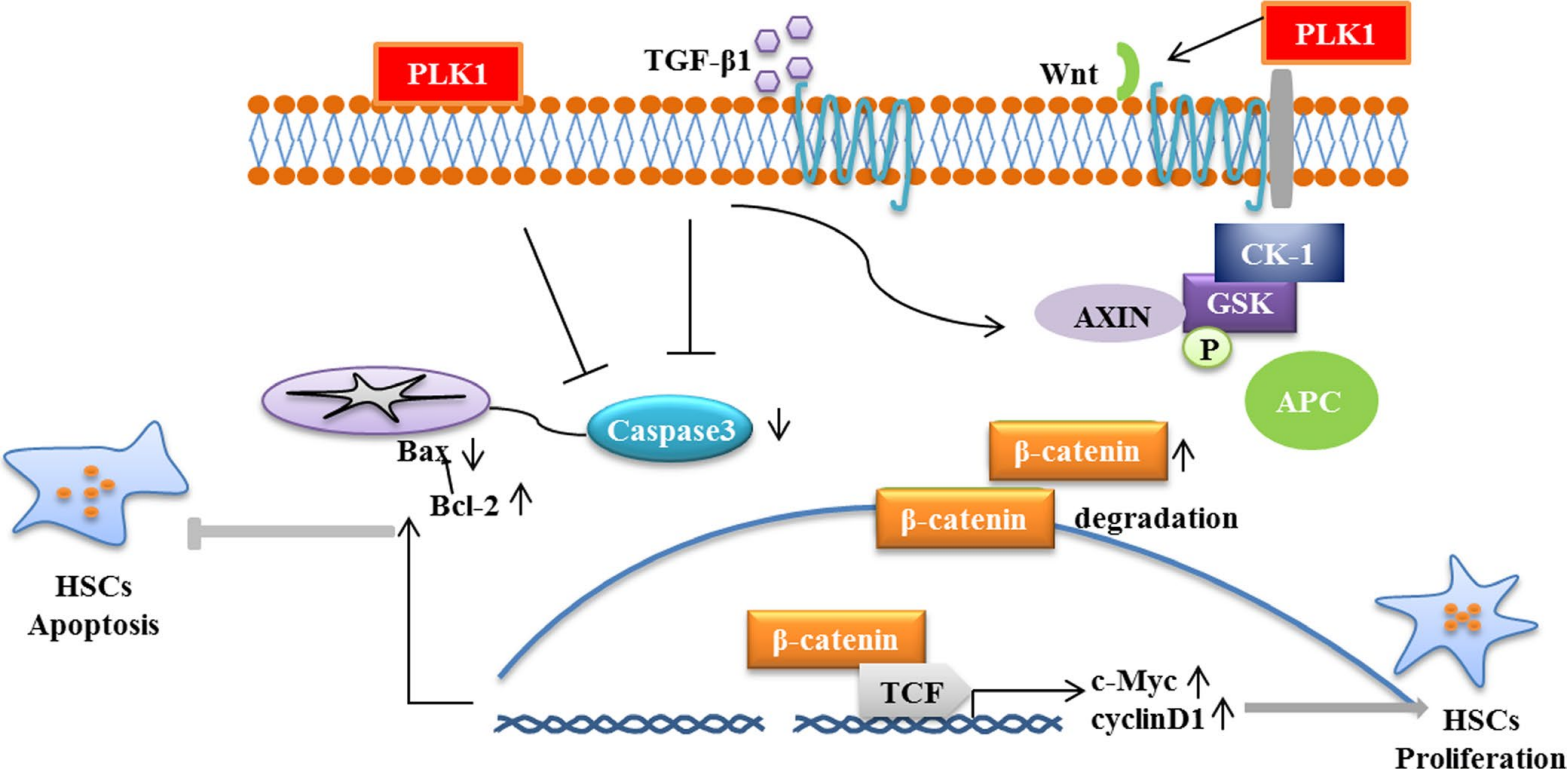
PLK1 regulates hepatic stellate cell activation and liver fibrosis through Wnt/β‐catenin signalling pathway

Previous studies have demonstrated that the Wnt/β‐catenin signalling pathway is involved in HSCs proliferation. The present study questioned whether PKL1 activates the Wnt/β‐catenin signalling pathway to regulate HSCs proliferation. We found that low expression of PLK1 by AAV‐shPLK1 significantly decreased the expression of β‐catenin, c‐Myc and Cyclin D1 in CCl_4_‐induced liver fibrosis mice compared with AAV‐empty mice (Figure 6A). Similarly, treatment of LX‐2 cells with Cas9‐gRNA‐PLK1 significantly reduced the expression of β‐catenin, c‐Myc and Cyclin D1 compared with the Cas9‐empty group (Figure 6B). Moreover, PLK1 overexpression by GTP‐PLK1 increased β‐catenin, c‐Myc and Cyclin D1 expression in LX‐2 cells compared with the GTP‐empty plasmid‐transfected group (Figure 6C). We further investigated the effect of PLK1 on the cell cycle of LX‐2 cells by flow cytometry. Inhibition of PLK1 induced cell cycle arrest in G0/G1 in LX‐2 cells stimulated with TGF‐β1 (Figure 6D). In contrast, PLK1 overexpression advanced LX‐2 cells stimulated with TGF‐β1 into G2/M compared with the GTP‐empty group by flow cytometry analysis (Figure 6E). Additionally, we used a TGF‐β1 inhibitor to block TGF‐β1 signalling. As shown in Figure S3A,B, the expression of PLK1 was significantly decreased in LX‐2 cells treated with TGF‐β1 inhibitor. These results indicate that PLK1 promoted the proliferation of HSCs by regulating the Wnt/β‐catenin signalling pathway in liver fibrosis in vivo and in vitro (Figure 7).

## 4 DISCUSSION

Liver fibrosis is an important global concern, and there is currently no approved treatment.^22^ It is a reversible pathological process in which an imbalance between the synthesis and degradation of the ECM leads to abnormal deposition of fibrous connective tissue in the liver.^23^ The activation, proliferation and transformation of HSCs caused by liver injury are important factors in the process of liver fibrosis.^24^ However, the molecular mechanisms by which HSCs are activated to induce fibrosis have not been fully elucidated.^25^ In our study, we found that increased PLK1 expression was associated with liver fibrosis and generated reliable data to support the pro‐fibrosis effect of PLK1. We demonstrated that PLK1 was increased in human liver fibrosis samples and primary HSCs from CCl_4_‐induced fibrosis mice using Western blot and real‐time PCR, whereas immunofluorescence and IHC assays further showed PLK1 was up‐regulated in the cytoplasm. In addition, we demonstrated that the up‐regulation of PLK1 in HSCs induced the expression of α‐SMA, Col1α1 and TIMP‐1 and promoted the activation of HSCs. We also found that the induction of PLK1 expression in HSCs further promoted the proliferation and activation of HSCs by increasing the expression of specific transcription factors associated with the Wnt/β‐catenin signalling pathway. In CCl_4_‐induced liver fibrosis mice, we found that reduced expression of PLK1 by AAV‐shPLK1 significantly decreased liver fibrosis. Our results suggest that PLK1 may be a potential therapeutic target for liver fibrosis.

PLK1 is a highly conserved serine/threonine kinase.^26^ Recent studies have revealed that PLK1 has a broad regulatory role in cell mitosis and plays a critical role in cell proliferation.^27^, ^28^ It has been confirmed that PLK1 is overexpressed in many different tumour types and thus has a critical effect on tumour development.^12^, ^29^ We found that inhibition of PLK1 expression could reduce the activation of HSCs and thus alleviate liver fibrosis. Moreover, inhibition of PLK1 could reduce the activation of the Wnt/β‐catenin signalling pathway in vivo and down‐regulate the expression of β‐catenin, c‐Myc and Cyclin D1 to block the proliferation of HSCs. Similarly, low expression of PLK1 by Cas9‐gRNA‐PLK1 lentivirus down‐regulated the expression of cell cycle‐related proteins in TGF‐β1–stimulated LX‐2 cells in vitro. In contrast, PLK1 overexpression by GTP‐PLK1 plasmid increased the expression of cell cycle‐related proteins β‐catenin, c‐Myc and Cyclin D1 in TGF‐β1–stimulated LX‐2 cells in vitro. Interestingly, inhibition of TGF‐β1 signalling decreased PLK1 expression, suggesting that a PLK1‐mediated Wnt/β‐catenin signalling axis plays an important role in the process of liver fibrosis by enhancing the activation of HSCs by TGF‐β1. Additionally, we observed that inhibition of PLK1 expression significantly increased HSCs apoptosis. We identified that PLK1 inhibition activated apoptotic executive proteins caspase‐3 and increased the Bax/Bcl‐2 ratio to promote the apoptosis of HSCs. Similarly, knockdown of PLK1 by Cas9‐gRNA‐PLK1 lentivirus up‐regulated the expression of apoptosis‐related proteins and increased the Bax/Bcl‐2 ratio in LX‐2 cells. In addition, PLK1 overexpression by PLK1‐GTP plasmid decreased the expression of cleaved caspase‐3 and the Bax/Bcl‐2 ratio in TGF‐β1–stimulated LX‐2 cells in vitro. Furthermore, the results of flow cytometry showed that inhibition of PLK1 expression increased the number of apoptotic LX‐2 cells. Considering this combined effect, PLK1 promoted the activation and proliferation of HSCs and inhibited apoptosis in liver fibrosis.

We conclude that PLK1 is an important molecule for HSCs activation in liver fibrosis. We identified low expression of PLK1 in liver fibrosis. Down‐regulated PLK1 in HSCs further alleviated the expression of proteins associated with HSCs proliferation (c‐Myc and Cyclin D1) and enhanced the expression of apoptosis‐related proteins (cleaved caspase‐3 and Bax/Bcl‐2). The current study demonstrates that inhibition of PLK1 expression prevents HSCs activation and proliferation to reduce liver fibrosis through the Wnt/β‐catenin signalling pathway. Our present study suggests that PLK1 is an attractive novel therapeutic target for liver fibrosis.

## CONFLICT OF INTEREST

The authors declare no conflict of interest.

## AUTHOR CONTRIBUTIONS

YC and XC conceived and designed the study. YC, XC and SZ performed the experiments. FTB collected and coordinated the data. YRJ provided the mouse. XSD and YC wrote the manuscript. XMM, CH and JL reviewed and modified the manuscript. All authors read and approved the manuscript.

## Supporting information

Figure S1

Figure S2

Figure S3

Supplementary Material

## Data Availability

The data used to support the findings of this study are included within the article.
