# Supplementary figures and images for: PLK1 regulates hepatic stellate cell activation and liver fibrosis through Wnt/β‐catenin signalling pathway
Source: J Cell Mol Med. 2020 May 28;24(13):7405–16. doi: 10.1111/jcmm.15356 (PMC7339205; doi:10.1111/jcmm.15356)

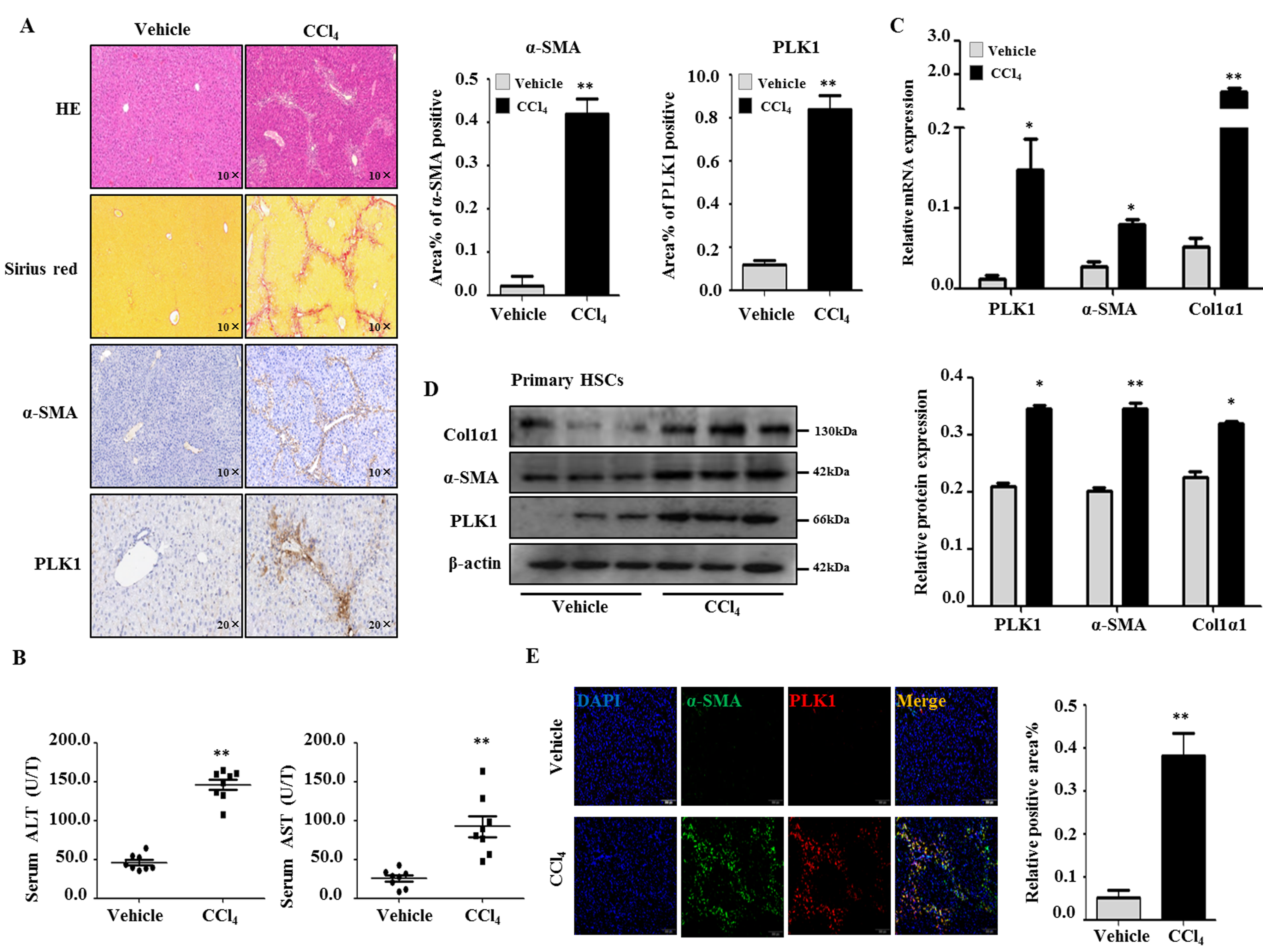

Supplement: Supplementary file 1 — Figure S1 [file JCMM-24-7405-s001.tif]

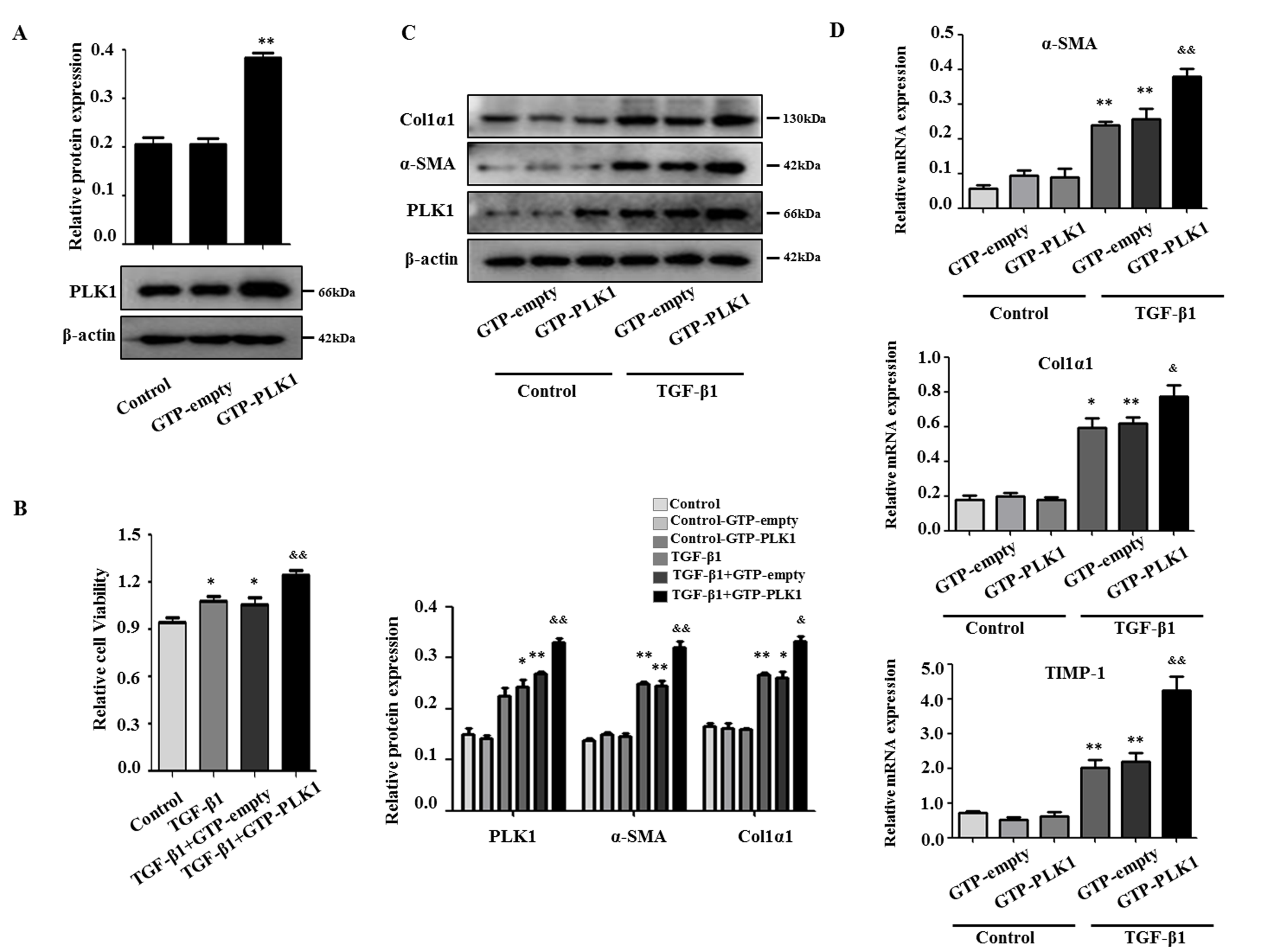

Supplement: Supplementary file 2 — Figure S2 [file JCMM-24-7405-s002.tif]

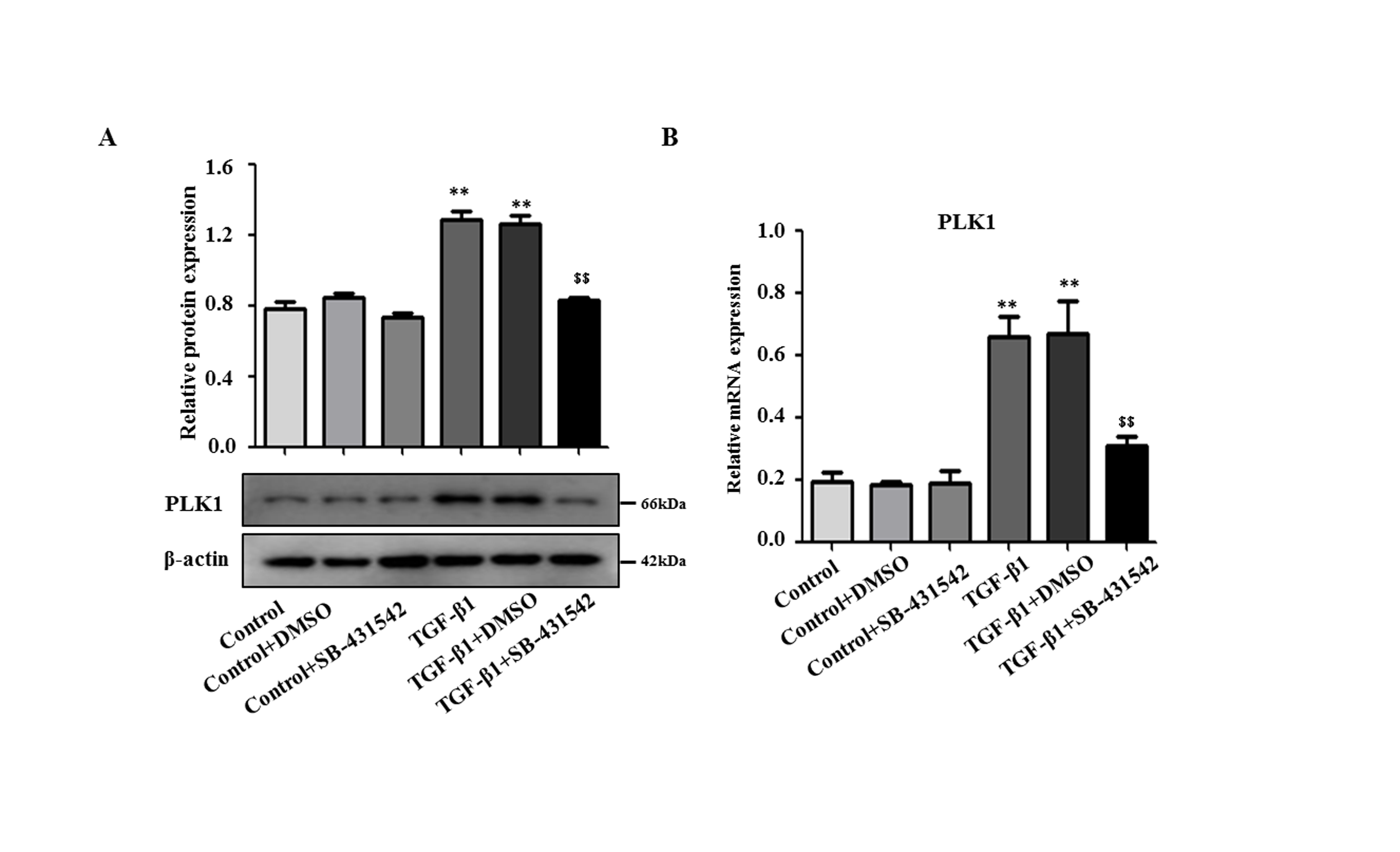

Supplement: Supplementary file 3 — Figure S3 [file JCMM-24-7405-s003.tif]
