# Supplementary material for: PLK1 regulates hepatic stellate cell activation and liver fibrosis through Wnt/β‐catenin signalling pathway
Source: J Cell Mol Med. 2020 May 28;24(13):7405–16. doi: 10.1111/jcmm.15356 (PMC7339205; doi:10.1111/jcmm.15356)
Supplement: Supplementary file 4 — Supplementary Material [file JCMM-24-7405-s004.docx]

**Supplementary** **Figure legends**

**Supplementary Figure 1. Upregulation of PLK1 expression in HSCs is associated with CCl_4_-induced liver fibrosis.** (A) Hematoxylin and eosin (H&E), Sirius red staining, and immunohistochemistry (IHC) were performed in samples from CCl_4_- and vehicle-treated mice. Representative images are shown. Scale bars, 100 µM and 50 µM. Quantification of fibrosis based on immunohistochemistry analysis of α-SMA and PLK1. (B) Serum ALT and AST levels were determined. (C) Fold changes in α-SMA, Col1α1, and TIMP-1 mRNA levels were shown by real-time PCR in primary HSCs isolated from vehicle and CCl_4_-treated mice. (D) The protein expression of PLK1, α-SMA, and Col1α1 was analyzed by western blotting of primary HSCs isolated from vehicle and CCl_4_-treated mice. (E) Representative double immunofluorescence staining of PLK1 (red) and α-SMA (green) in vehicle and CCl_4_-treated mouse liver are presented. Scale bar, 100 µM. Quantitative data of positive co-localization areas are shown. The data represent the mean ± SEM of at least three independent experiments. ******P*<0.05, *******P*<0.01 *vs.* vehicle group.

**Supplementary Figure 2. Overexpression of PLK1 promotes the activation of LX-2 cells stimulated with TGF-β1.** (A) Western blot analyses of PLK1 in LX-2 cells transfected with GTP-PLK1 or Control-empty. (B) Relative cell viability of LX-2 cells was measured by CCK-8 assay. (C) Western blot analyses of PLK1, α-SMA, and Col1α1, (D) and mRNA levels of α-SMA, Col1α1, and TIMP-1 in LX-2 cells stimulated with TGF-β1 following GTP-PLK1. ******P*<0.05, *******P*<0.01 *vs.* control; **^&^***P*<0.05, **^&&^***P*<0.01 *vs.* TGF-β1+GTP-empty. Data shown are the mean ± SEM of three independent experiments.

**Supplementary Figure 3.** **Inhibition of TGF-β1 signaling decreased PLK1 expression.** (A) Western blot analyses of PLK1 in LX-2 cells transfected with SB-431542 (TGF-β1 inhibitor). (B) mRNA levels of PLK1 in LX-2 cells following SB-431542.
